# Model-based and model-free decision making in major depressive disorder after performing behavioral training

**DOI:** 10.1192/j.eurpsy.2022.593

**Published:** 2022-09-01

**Authors:** A. Bampi, C. Sorg, F. Brandl

**Affiliations:** Neuro-Kopf-Zentrum TU Munich, Neuropsychiatry And Neuroimaging Lab, Munich, Germany

**Keywords:** behavioral training, decision making, model based, major depressive disorder

## Abstract

**Introduction:**

In major depressive disorder (MDD), reward-based decision-making (DM) is frequently impaired: e.g. patients don’t engage in pleasant activities as much as healthy subjects. Put differently, previous and expected future rewards have less reinforcing effects on DM. This study investigated two experimentally well-observable reward-based DM modes, namely model-based (based on cognitive models of the environment) and model-free (based on previous experience) DM.

**Objectives:**

We hypothesized that model-based training can improve reward-based DM in patients with MDD. Answers to these questions could enhance the development of cognitive-behavioral therapeutic interventions.

**Methods:**

27 patients with MDD were recruited and assessed with psychometry. All patients performed the „two-step Markov decision-task“ (Daw, 2011), which allows the simultaneous investigation of model-based and model-free DM via computational modelling. All subjects performed the task 4 times: at the beginning and at the end of 2 assessment days (session-interval: 4 days). Subjects were randomly allocated to an intervention group, which performed model-based training, and a control group, which performed model-free training. The main outcomes of training effect were the influence of model-based reward expectations on decisions (quantified by computational modelling parameters) and overall monetary reward-success.

**Results:**

In all patients, the influence of model-based reward expectations on decisions increased after training. However, there was no significant effect of group allocation. Furthermore, patients in the intervention group did not achieve significantly higher overall monetary reward.

**Conclusions:**

Results suggest that in MDD, the influence of model-based reward expectations on decisions can be improved regardless of specific training type. Future studies should investigate the effects on everyday functioning.

**Disclosure:**

No significant relationships.

